# Establishment of the European meningococcal strain collection genome library (EMSC-GL) for the 2011 to 2012 epidemiological year

**DOI:** 10.2807/1560-7917.ES.2018.23.20.17-00474

**Published:** 2018-05-17

**Authors:** Holly B Bratcher, Carina Brehony, Sigrid Heuberger, Despo Pieridou-Bagatzouni, Pavla Křížová, Steen Hoffmann, Maija Toropainen, Muhamed-Kheir Taha, Heike Claus, Georgina Tzanakaki, Tímea Erdôsi, Jelena Galajeva, Arie van der Ende, Anna Skoczyńska, Marina Pana, Alena Vaculíková, Metka Paragi, Martin CJ Maiden, Dominique A Caugant

**Affiliations:** 1 University of Oxford, Department of Zoology, Oxford, United Kingdom; 2 Clinical Science Institute, National University of Ireland, Galway, Republic of Ireland; 3 Austrian Agency for Health and Food Safety, Graz, Austria; 4 Nicosia General Hospital, Nicosia, Cyprus; 5 National Institute of Public Health, Prague, Czech Republic; 6 Statens Serum Institute, Copenhagen, Denmark; 7 National Institute for Health and Welfare, Helsinki, Finland; 8 Pasteur Institute, Paris, France; 9 University of Würzburg, Würzburg, Germany; 10 National Meningitis Reference Laboratory, Athens, Greece; 11 National Center for Epidemiology, Budapest, Hungary; 12 Riga East University Hospital, Riga, Latvia; 13 Academic Medical Center, University of Amsterdam, The Netherlands; 14 National Medicines Institute, Warsaw, Poland; 15 National Institute of Research and Development for Microbiology and Immunology, Bucharest, Romania; 16 Public Health Authority of the Slovak Republic, Bratislava, Slovakia; 17 National Institute of Public Health, Ljubljana, Slovenia; 18 Norwegian Institute of Public Health, Oslo, Norway

**Keywords:** *Neisseria meningitidis*, surveillance, genome library, monitor vaccine coverage, track antimicrobial susceptibility

## Abstract

Invasive meningococcal disease surveillance in Europe combines isolate characterisation and epidemiological data to support public health intervention. A representative European Meningococcal Strain Collection (EMSC) of IMD isolates was obtained, and whole genome sequenced to characterise 799 EMSC isolates from the epidemiological year July 2011–June 2012. To establish a genome library (GL), the isolate information was deposited in the pubMLST.org/neisseria database. Genomes were curated and annotated at 2,429 meningococcal loci, including those defining clonal complex, capsule, antigens, and antimicrobial resistance. Most genomes contained genes encoding B (n = 525; 65.7%) or C (n = 163; 20.4%) capsules; isolates were genetically highly diverse, with >20 genomic lineages, five of which comprising 60.7% (n = 485) of isolates. There were >350 antigenic fine-types: 307 were present once, the most frequent (P1.7-2,4:F5-1) comprised 8% (n = 64) of isolates. Each genome was characterised for Bexsero Antigen Sequence Typing (BAST): 25.5% (n = 204) of isolates contained alleles encoding the fHbp and/or the PorA VR1 vaccine component, but most genomes (n = 513; 64.2%) did not contain the NadA component. EMSC-GL will support an integrated surveillance of disease-associated genotypes in Europe, enabling the monitoring of hyperinvasive lineages, outbreak identification, and supporting vaccine programme implementation.

## Introduction

Invasive meningococcal disease (IMD) is a contagious, occasionally epidemic, infectious disease that causes significant morbidity and mortality with varying incidence globally [1]. Only a small proportion of individuals infected with *Neisseria meningitidis*, the meningococcus, develop severe disease, usually meningitis and/or septicaemia, with survivors often suffering permanent sequela [2]. While vaccination with protein-conjugated polysaccharides of serogroup A, C, W, and Y is an effective prevention strategy for the main disease-causing serogroups, currently there is no comprehensive vaccine against meningococcal disease. The structural similarity of the serogroup B polysaccharide capsule to the human neural cell adhesion molecule renders it poorly immunogenic and carries concerns over possible autoimmune reactions [3,4]. To circumvent this problem, vaccines developed to control serogroup B, the main cause of IMD in Europe, have been based on outer-membrane vesicles and more recently on recombinant proteins [5,6].

Understanding the impact of meningococcal vaccines requires surveillance that includes knowledge of disease-associated meningococci and the antigens and capsule types that they possess. However, comprehensive and timely surveillance of IMD is complicated by the high genetic and antigenic diversity of meningococcal populations [7]. While most meningococcal disease is caused by a minority of meningococcal capsule types, there are many variants of the subcapular antigens that are used in vaccines [8]. However, relatively few meningococcal genotypes, known as hyperinvasive lineages which have been identified by multilocus sequence typing (MLST) as particular clonal complexes (ccs), cause most IMD and these have been shown to be associated with particular variants of antigens and combination of vaccine antigen variants [7,9]. Conventional isolate typing has required multiple tests involving sequencing of multiple genes [10] but whole genome sequencing (WGS) provides the potential to identify all of the salient features in a single experiment [11].

The establishment of a European meningococcal genome library has potential to enhance IMD surveillance continent-wide, providing comprehensive and comparable long-term typing data. To do this, all national reference laboratories for meningococci in Europe were asked to make available in 2014 isolates from one epidemiological year (2011–2012) to contribute to the European Meningococcal Strain Collection (EMSC) in order to provide a bank of isolate genomes representative of those causing invasive disease in Europe at that time. The EMSC Genome Library (EMSC-GL) was created to exploit whole genome sequence (WGS) data for surveillance, characterisation, and vaccine intervention studies to analyse longitudinal data using both historical, current and future datasets. The WGS data are hosted on the pubMLST.org/neisseria database*, *where the analysis tools of the BIGSdb platform can be used [12].

## Methods

### Specimen collection

The EMSC was established in 2002 at the Norwegian Institute of Public Health, Oslo, Norway with the support of the European Centre for Disease Prevention and Control (ECDC), to assure preservation of meningococcal isolates causing disease in Europe. Additional funding was allocated by the ECDC in 2011 for the implementation of the EMSC-GL. Participation of each country in the creation of the EMSC-GL was voluntary. All 799 invasive meningococcal isolates received from the epidemiological year July 2011–June 2012, from 16 participating national reference laboratories, were included in the EMSC-GL.

### Culture and DNA extraction

Isolates were grown on blood agar plates overnight at 37 °C in an atmosphere of 5% CO_2_ and DNA extracted using an epMotion 5075 pipetting robot (Eppendorf) and NucleoSpin 8 Tissue kit (Macherey-Nagel) according to the manufacturer’s instructions. DNA was visualised with the FlashGel DNA system (Lonza), its quantity assessed using Qubit (Invitrogen, ThermoFisher Scientific Inc), and sent frozen in 96-well plates to the Oxford Genomics Centre (OGC), University of Oxford, United Kingdom (UK).

### Whole genome sequencing, de novo assembly, annotation and curation

The DNA samples were normalised and sequenced at the OGC. Quantification and integrity of DNA extracts were assessed using the Invitrogen PicoGreen assay on a 1% E-GelEX. DNA was fragmented using acoustic shearing and the fragment distribution determined using the Agilent Tapestation D1200 system. NEBNext DNA Sample Prep Master (New England BioLabs) was used with minor modifications to construct the libraries. Ligation of the adapters was completed using Illumina Adapters and PCR enriched. Libraries were pooled and quantified using with the MX3005PTM instrument (Agilent) and the Agilent qPCR Library Quantification Kit. Paired-end 100 base reads were sequenced using the Illumina HiSeq 2000 sequencing platform.

Isolate records were created in the pubMLST.org/neisseria database and included provenance and phenotypic typing information [12]. Short-read sequence data were assembled *de novo* as described previously [13], and, uploaded to the database and then linked to their respective isolate record. Genome assembly statistics were generated as part of this automated pipeline. The data are accessible on pubMLST.org/neisseria web page.

Each genome assembly was queried against the pubMLST.org/neisseria sequence definition database to identify the loci within individual genomes. As identified, loci were automatically annotated with a *Neisseria* (NEIS) locus identifier and an allele number assigned if an identical reference sequence was present in the database. Novel alleles with ≥ 98% sequence identity to a reference sequence in the database, were automatically assigned new reference numbers. This process was repeated until all NEIS loci had been curated and as many alleles as possible had been defined and assigned in each genome assembly. The new alleles with < 98% sequence identity were manually checked and curated before assigning a new reference number. The annotated loci included those defining antigens, genes known to effect antibiotic sensitivity, the capsule operon genes, and the 1,605 loci belonging to the N. meningitidis core genome MLST (cgMLST) scheme (version 1.0) [9,13,14]. Alleles with a known antibiotic sensitivity range were previously submitted to the pubMLST.org/neisseria sequence definition database and EMSC-GL alleles with the same sequence mutation pattern were inferred to convey the same antibiotic sensitivity range [15-17].

### Genomic analysis and population structure

Using the ‘sequence bin breakdown’ tool, the assembly statistics and genome coverage were assessed for each genome assembly. The BIGSdb Genome Comparator Tool was used to generate allele-based pairwise comparison distance matrices that were visualised as Neighbour-Net phylogenies. Cramer’s V coefficient was used to measure the association of clonal complex (cc) with Bexsero Antigen Sequence Typing (BAST) and individual vaccine antigens, using the ‘cramersV’ function in the ‘lsr’ package in R 3.3.0 [18]. The coefficient ranges from zero to one, with zero indicating no association and one indicating complete association.

### Curatorial support and database operation

The data confidentiality and security: no personal identification is recorded in the database and isolates are identified by an anonymised sample name and an automatically assigned isolate accession number. Each reference laboratory is provided immediate access to their submitted data once it has been received and assessed by a curator. The pubMLST database and its curators are currently supported by The Wellcome Trust (grant 104992) and the UK Department of Health (grant H2R00080) respectively.

## Results

### Assembly and genetic coverage

The isolates (n = 799) represented IMD cases from 16 EU countries during the epidemiological year July 2011–June 2012. The genome assemblies contained an average of 466 contigs, with an average length of 5,211 nt per contig, and an average genome length of 2,143,632 nt. The larger number of contigs and unassembled genes compared to other studies was due to fragmentation of the DNA during extraction process [13,19]. The pubMLST.org/neisseria database (accessed 06/2016) contained 2,429 curated loci, of which 1,605 genes (66.1%) are considered to be a part of the core genome (cgMLST, v1.0) [13]. The curated loci are composed of coding sequences, typing fragments, antigenic peptides, intergenic promotor regions, and other sequence regions of interest. Each draft genome contained between 1,724 and 1,933 identifiable genes with an average of 1,831 per genome. The average genetic content for a *N. meningitidis* genome, based on finished genomes (NZ-05/33, FAM18, MC58, H44/76, Z2491, F2136 and M01–240196), was 1,967 genes.

### Capsule

Isolates had been tested for capsule phenotype by the submitting laboratory: 65.8% were serogroup B (n = 526); 20.8% were serogroup C (n = 166); 7.5% were serogroup Y (n = 60); 4.0% were serogroup A, W, or W/Y (n = 1; 30; 1); and 1.9% were non-groupable (NG) or had no serogroup data recorded (n = 14; 1). Analysis of capsule operon regions A and C from the WGS data: (i) genotypically confirmed the phenotypic serogrouping of 767/799 isolates (96.0%); (ii) defined the genogroup of 14 NG isolates and of one isolate without serological data; (iii) corrected the capsule designation of 14 (1.8%) isolates; and (iv) partially confirmed one B and one C capsule and found one mosaic capsule operon (Table 1).

**Table 1:** Clarification of capsule type of European Meningococcal Strain Collection isolates using whole genome analysis, July 2011– June 2012 (n = 32 isolates)

Capsule operon region A and C genes	Number of isolates	Serogroup result
C	5	B
E	1	
Cnl	1	
Mosaic B(+E)	1	
Incomplete assembly	1	
	1	C
Y	1	
B	2	
	1	ND
	3	Y
	5	NG
Cnl	2	
E	1	
W	2	
W/Y	1	
X	2	
Y	1	
	1	W

Cnl: capsule null; mosaic B (+ E): complete B operon plus partial E found; ND: not done; NG: non-groupable.

Assignment of capsule type using gene-by-gene analysis of capsule operon in each genome where serogroup testing did not correspond or was not done; including one mosaic capsule operons (IBD-1081, B with partial E). Two genomes with non-groupable serotype results (IBD-407, B; IBD-409, B) were found to be genetically phase variable off.

The isolate without serological typing information contained capsule type B genes. Two genomes had a poorly assembled region A and the gene that determines capsule type was incompletely assembled; however, the assembled sequence indicated that genetically one isolate was B and one isolate was C. 

Of the serogroup records that did not match capsule genotype: three isolates assigned to serogroup C were capsule type B (n = 2) and capsule type Y (n = 1); three serogroup Y were capsule type B; seven isolates assigned to serogroup B were capsule type C (n = 5), capsule type E (n = 1), and one isolate did not contain the capsular regions A or C and was confirmed to be a capsule null (*cnl*); and one typed as serogroup W was genetically a capsule type Y. 

The capsule types determined for the NG isolates included: five capsule type B; two capsule type W; two capsule type X; one capsule type E; two *cnl*; one capsule Y; and one with a serine residue at codon 310 of the polymerase gene, which would result in a mixed sialic acid capsule phenotype for W and Y [20]. Two of the NG isolates contained capsule B genes but were genetically phase variable off. 

The isolate with a mosaic capsule contained capsule biosynthesis genes from serogroup B in addition to a partial region A for capsule type E. There was no report of secondary capsule expression nor inconclusive serogroup results reported for the isolate. 

### Clonal complex

MLST was determined from the WGS data. For 402/799 isolates (50.3%) MLST data obtained by conventional means were also available. The WGS data confirmed the sequence type (ST) and cc for 377/402 (93.8%) of these isolates and determined the ST and cc for 395 of the remaining 397 (99.5%) isolates. 

Concerning the 25 isolates among the 402, where the conventional MLST and the WGS data did not concur: (i) for two the *gdh* MLST locus was incomplete but the remaining six MLST alleles were identified: one isolate had a profile consistent with the ST-269cc, but was nonetheless not assigned to any specific ST, while the other was unique and did not match any currently defined ST; (ii) and for 23 isolates, the WGS-derived MLST differed from that previously reported. Most (17/23) differed at a single MLST locus (including 10 genomes differed at *pgm*, 4 at *fumC*, 2 at *abcZ*, and 1 at the *aroE* locus), three differed at two loci (*pgm* and *abcZ* or *fumC* or *gdh,* respectively), and three isolates differed at all seven loci. The isolates where the ST was one or two loci different were still assigned to the same cc, the three isolates with seven loci differences were each reassigned to a different cc. These results were consistent with the downstream effects of splitting samples in seven-locus PCR-based MLST and mislabelling of samples [13]. 

**Figure 1:**
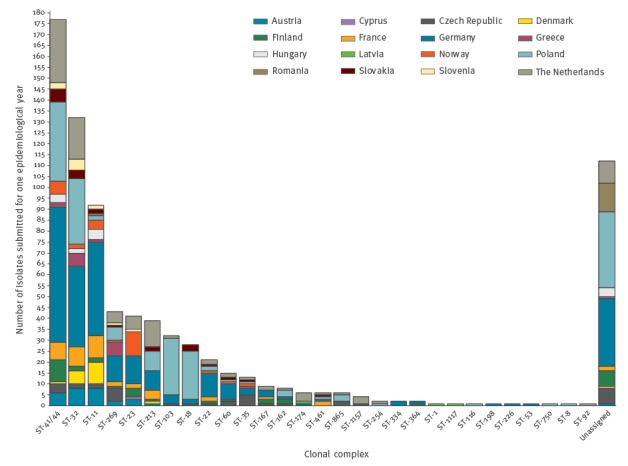
Breakdown by clonal complex of invasive meningococcal disease isolates submitted by 16 European countries for the epidemiological year July 2011–June 2012 (n = 799 isolates)

In total, 795 isolates had an assigned ST, among which 108 with no cc association. Two-hundred-ninety STs were defined (795/799; 99.5%) and twenty-nine cc were identified (687/799; 86.0%). The three most common cc, cc41/44 (n = 177 isolates), cc32 (n = 132), and cc11 (n = 92), represented 50.2% of the 799 isolates. Eight additional cc had at least 10 representatives each and comprised 29.0% (232) of the isolates (Figure 1). The remaining 18 cc were represented by one to nine isolates each. The 108 isolates (13.5%) without a cc association were less than 1% of the 290 STs defined. Overall the most prominent STs were ST-5238 from Poland (n = 6) and Germany (n = 1) and ST-10958 from Romania (n = 8).

ST: sequence type.

The most commonly observed were clonal complexes for the epidemiological year were ST-41/44, ST-32 and ST-11. The isolates unassigned to a clonal complex included four isolates without an ST and 108 isolates with a ST that was not associated with a clonal complex.

### Antigenic typing and vaccine coverage

The antigenic fine-type data (PorA VR1:PorA VR2:FetA VR) were assessed for each of the isolates’ genome assemblies. The finetype antigen profile was complete in 778 of the isolates (97.4%). The *porA* gene (NEIS1364) was absent in 10 genomes (1.3%), while four genomes had a deleted PorA VR1 loop, two genomes had a deleted PorA VR2 loop, and one genome had an incompletely assembled NEIS1364 gene and its PorA VR1 loop could not be assigned. Additionally, six genomes (0.8%) were lacking FetA VR assignment: five were missing the NEIS1963 (*fetA*) gene and one had a non-coding protein sequence. The most prevalent antigen fine-types in the dataset (n ≥ 10) included: 7–2:4:F1–5 (8.0%); 7:16:F3–3 (5.2%); 5:2:F3–3 (4.7%); 22:14:F5–5 (3.7%); 18–1:3:F3–9 (3.1%); 5–1:10–8:F3–6 (3.0%); and 5–2:10–1:F4–1 (3.0%).

**Figure 2:**
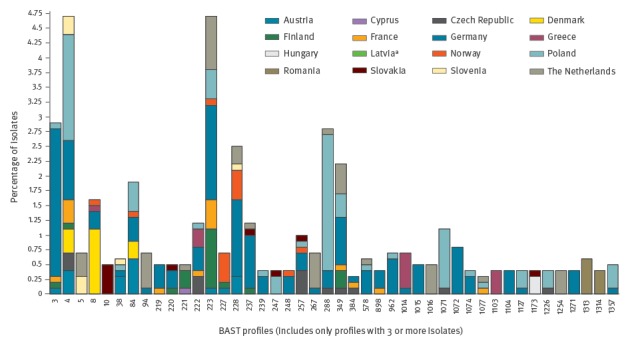
Putative vaccine antigen coverage of invasive meningococcal disease isolates, as assessed by a direct sequence match to any one of four vaccine components, Europe, July 2011–June 2012 (n = 349 isolates^a^)

The meningococcal genomes were characterised with the BAST scheme. The 4CMenB (Bexsero) vaccine contains four different peptide components and is represented by the peptide profile BAST-1 (fHbp 1, NHBA 2, NadA 8, PorA VR1 7–2, PorA VR2 4) [9]. There were no occurrences of BAST-1 in the EMSC-GL. BAST antigens were diverse and 687 genomes (86.0%) were assigned to one of 332 BASTs. The genomes without a BAST assigned (n = 112; 14.0%) had an incomplete sequence assembly in the region containing the NEIS2109 (*nhba) *or the NEIS1969 (*nadA)* gene. The most common 12 BASTs were present at a frequency of at least 1.0% (n = 8) and an additional 32 BASTs were represented by at least three to seven genomes each (Figure 2).

BAST: Bexsero Antigen Sequence Type.

The vaccine components are NadA, NHBA, PorA VR1 and PorA VR2.

^a^ The graph depicts the 44 BASTs found with three or more examples, a total of 332 BAST were found in the dataset. Putative multiple component coverage BAST include: three exact sequence match vaccine components (BAST-223); two exact sequence match vaccine components (BAST-84, 2448, 1071, 1072, and 1077); and one exact sequence match vaccine component (BAST-4, 5, 10, 94, 239, 1014, 1016, 1074, 1271, and 1313).

Among the total 332 BASTs, the remaining BASTs included 50 profiles (15.1%) with two representative genomes each and 238 profiles (71.7%) that occurred once. Overall the two most common profiles were BAST-223 (FHbp 14, NHBA 2, NadA 0, PorA VR1 7–2, PorA VR2 4) and BAST-4 (FHbp 1, NHBA 3, NadA 1, PorA VR1 7, PorA VR2 16). The gene encoding *nadA* was absent in 64.2% (n = 513) of 799 genomes and 229 of the BASTs. The BAST of each genome was assessed for direct matches to the BAST-1 components for prediction of potential vaccine coverage. Predicted coverage, based on sequence identity, was most frequently associated with the PorA VR1 7–2 (n = 116; 14.5%) and fHbp 1 (n = 113; 14.1%) vaccine components (Table 2).

**Table 2:** Putative vaccine antigen coverages of invasive meningococcal disease isolates, based on sequence matching using the Bexsero antigen sequence type (BAST) scheme and assessing presence of the fHbp peptide components in the bivalent rLP4086 (Trumemba), Europe, July 2011–June 2012 (n = 799 isolates)

European country	Number of isolates in dataset	NadA peptide 8	NHBA peptide 2	FHbp peptide 1	PorA VR1 peptide 7-2	PorA VR2 peptide 4	BAST total (%)	FHbp peptide 45	FHbp peptide 55	Bivalent total (%)
Austria	30	0	1	9	7	1	18 (60)	1	0	1 (3.3)
Cyprus	1	0	0	0	0	0	0 (0.0)	0	0	0 (0.0)
Czech Republic	36	0	1	2	3	0	6 (16.7)	0	0	0 (0.0)
Denmark	19	0	0	6	3	0	9 (47.4)	1	0	1 (5.3)
Finland	34	0	10	2	11	10	33 (97.1)	1	0	1 (2.9)
France	42	0	4	6	6	6	22 (52.4)	4	0	4 (9.5)
Germany	245	1	41	26	45	36	149 (60.8)	3	0	3 (1.2)
Greece	17	0	0	6	0	0	6 (35.3)	0	0	0 (0.0)
Hungary	15	0	1	1	1	0	3 (20.0)	0	0	0 (0.0)
Latvia	3	0	0	0	0	0	0 (0.0)	0	0	0 (0.0)
Norway	27	0	3	2	5	3	13 (48.2)	0	0	0 (0.0)
Poland	181	0	14	26	15	15	70 (38.7)	7	0	7 (3.9)
Romania	14	8	0	0	0	0	8 (57.1)	0	0	0 (0.0)
Slovakia	22	0	0	5	2	1	8 (36.4)	2	0	2 (9.1)
Slovenia	12	0	4	4	1	1	10 (83.3)	0	0	0 (0.0)
The Netherlands	101	3	16	18	17	10	64 (63.4)	8	0	8 (7.9)
All countries Total (%)	799 (100)	12 (1.5)	95 (11.9)	113 (14.1)	116 (14.5)	83 (10.4)	419 (52.4)	27 (3.4)	0 (0.0)	27 (3.4)

The Bexsero vaccine is composed of four different peptide components from four different clonal complexes and is represented by the peptide profile BAST-1 (fHbp 1, NHBA 2, NadA 8, PorA VR1 7–2, PorA VR2 4). The Trumemba vaccine is composed of two fHbp peptides, variants 45 and 55.

The genomes were also assessed for exact peptide matches for the bivalent rLP2086 (Trumemba) vaccine composed of fHbp variants 45 and 55. The fHbp variant 55 was not observed, while variant 45 was present in 27 (3.4%) genomes from eight countries: the Netherlands (n = 8), Poland (n = 7), France (n = 4), Germany (n = 3), Slovakia (n = 2), and one each in Austria, Denmark, and Finland. There was no overlap of coverage between the vaccines, 52.4% (n = 419) of the genomes had exact matches to BAST component and an additional 3.4% (n = 27) of the genomes contained the fHbp variant 45.

### Antimicrobial resistance

The pubMLST.org/neisseria database contained some phenotypic antimicrobial data in the form of minimum inhibitory concentration (MIC) ranges. These data were limited to a subset of isolates used for targeted gene sequencing to define susceptibility for ciprofloxacin (NEIS1320, *gyrA* gene), penicillin (NEIS1753, *penA* gene), and rifampicin (NEIS0123, *rpoB* gene) [15,16]. Alleles without MIC data were compared with those with a MIC value to determine if the allele sequence contained known mutations associated with a phenotypic MIC range (Table 3).

**Table 3:** Evaluation of putative antibacterial susceptibility of invasive meningococcal disease isolates by sequence comparison, Europe, July 2011–June 2012 (n = 799 isolates)*

Country	*gyrA* gene: ciprofloxacin^a^				*rpoB* gene: rifampicin^b^		*penA* gene: penicillin^c^				
	tS^d^	tS(I)^d^	eS^e^	UKN	tS^d^	UKN	tS^d^	tS(I)^d^	tI^d^	tS(I)^d^	UKN
Austria	16	13	0	1	30	0	0	3	15	1	11
Cyprus	1	0	0	0	1	0	0	0	0	0	1
Czech Republic	7	29	0	0	34	2	1	8	15	0	12
Denmark	6	13	0	0	19	0	0	0	17	0	2
Finland	10	12	12	0	31	3	0	6	15	1	12
France	14	26	0	2	41	1	0	9	20	1	12
Germany	84	154	0	7	240	5	0	25	141	7	72
Greece	6	10	0	1	17	0	0	2	13	0	2
Hungary	5	9	0	1	15	0	0	1	10	1	3
Latvia	2	1	0	0	3	0	0	0	3	0	0
Norway	11	9	7	0	26	1	0	6	12	0	9
Poland	41	86	52	2	175	6	1	36	102	5	37
Romania	5	9	0	0	14	0	0	12	2	0	0
Slovakia	10	10	0	2	21	1	0	3	11	1	7
Slovenia	7	5	0	0	12	0	0	1	6	0	5
The Netherlands	26	51	22	2	98	3	1	20	52	5	23
**Sensitive**	251	0	93	0	777	0	3	0	0	22	0
**Intermediate resistance**	0	437	0	0	0	0	0	132	434	0	0
**Unknown**	0	0	0	18	0	22	0	0	0	0	208

I: intermediate; MIC: minimum inhibitory concentration; S: susceptible; S(I): allele for which additional sensitive or intermediate MIC ranges (µg/mL) have been recorded; UKN: unknown.

Isolates in this study were not directly tested for a MIC but where a genome contained a known allele with a MIC assay result the isolate was interpreted as having the same antibiotic sensitivity value.

^a ^Ciprofloxacin MIC range in µg/mL: ≤ 0.03 (susceptible); > 0.03 – < 0.12 (intermediate); > 0.12 (resistant).

^b ^Rifampicin MIC range in µg/mL: ≤ 1 (susceptible); > 1 (resistant).

^c^ Penicillin MIC range in µg/mL:  ≤ 0.06 (susceptible); > 0.06 – 1 (intermediate); > 1 (resistant).

^d^ t: tested MIC (the European meningococcal strain collection genome library isolate allele is an exact sequence match to an allele previously submitted to the database with a MIC tested value).

^e^ e: expected MIC (the European meningococcal strain collection genome library isolate allele has key known mutations allowing to infer antibacterial susceptibility).

Forty-one *rpoB* alleles were present in the dataset, 23 of which were associated with a susceptible MIC (≤ 1µg/mL). Most genomes (n = 501; 62.7%) contained one of four *rpoB* alleles: 18, 2, 4, or 34, and an additional eight alleles were present in at least 1% of the genomes (n = 256; 32.0%). The next most prevalent, allele 184 (n = 5; 0.63%), had no MIC associated testing data and was found in Germany (n = 3) and Poland (n = 2). There were 18 additional alleles, each present in the dataset once, with no MIC associated testing data.

There were 141 genomes (17.7%) with an incompletely assembled *penA* gene and no allele assignment and 44 genomes (5.5%) did not have the *penA* gene. There were 67 *penA* alleles in the dataset, 13 of which had MIC values that were generally sensitive, ≤ 0.06µg/mL (two alleles were always sensitive and 11 alleles were sensitive but had at least one isolate record where the MIC value was intermediate, > 0.06 to 1µg/mL). Thirty-eight *penA* alleles were associated with MIC values of intermediate sensitivity (> 0.06 to 1µg/mL). There were 16 additional alleles with no MIC associated testing data, 15 of which were found once in the dataset and one allele was found eight times, from Germany (n = 4), Poland (n = 3) and Hungary (n = 1).

There were a total of 19 *gyrA* alleles and 642 genomes (80.4%) contained allele 4, 2, or 3. Eight genomes (1.0%) had an incompletely assembled *gyrA* gene. Five additional alleles were found in at least 1% of the isolate genomes and represented an additional 16.5% of the genomes (n = 132). All eight alleles had an associated susceptible MIC test result (≤ 0.03µg/mL) and half of the alleles had at least one isolate record where the MIC result was intermediate (> 0.03 but < 0.12µg/mL). Only three of the eleven remaining alleles had MIC associated data and sequence comparison of the eight alleles with known MIC data was not discriminatory.

### Population structure

**Figure 3:**
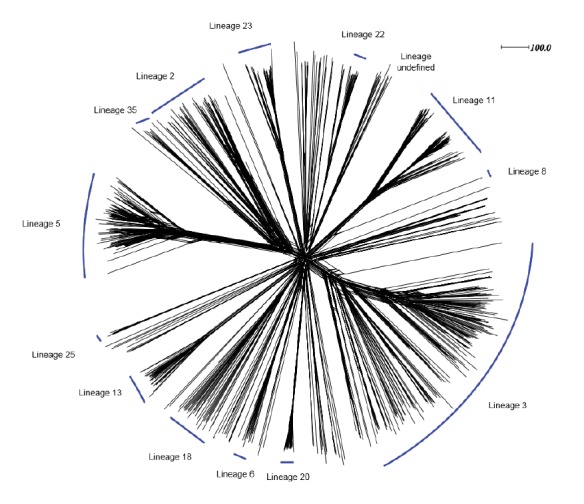
Neighbour-net population model analysis of invasive meningococcal disease isolates, Europe, July 2011–June 2012 (n = 799 isolates)

Meningococcal lineage nomenclature was used as the fundamental unit of analysis for the genetic relationships at the genome level [13]. This is based on ribosomal sequence types (rST) derived from the 53 ribosomal protein genes (rMLST) and supported with cgMLST analysis [13,21]. There were 788 complete rSTs, and 11 rSTs unassigned due to the incomplete assembly of one (n = 6), two (n = 3), or three (n = 2) of the 53 loci. A total of 13 lineages, corresponding to a known cc were identified: lineage 3 (cc41/44); lineage 8 (cc8); lineage 11 (cc11); lineage 22 (cc22); lineage 23 (cc23); lineage 2 (cc269); lineage 35 (cc35); lineage 5 (cc32); lineage 25 (cc162); lineage 13 (cc213); lineage 18 (cc18); lineage 6 (cc60); and lineage 20 (cc103) (Figure 3).

cc: clonal complex.

Core genome multilocus sequence typing (cgMLST, vr1) and lineage assignment were performed using the pubMLST Genome Comparator Tool and visualised with SplitsTree4. Correlation of cc and lineage assignment are as follows: lineage 3 (ST-41/44 cc), lineage 8 (ST-8 cc), lineage 11 (ST-11 cc), lineage 22 (ST-22 cc), lineage 23 (ST-23 cc), lineage 2 (ST-269 cc), lineage 35 (ST-35 cc), lineage 5 (ST-32 cc), lineage 25 (ST-162 cc), lineage 13 (ST-213 cc), lineage 18 (ST-18 cc), lineage 6 (ST-60 cc), lineage 20 (ST-103 cc). Unlabelled genomes are currently unassigned to a defined lineage but may be associated with a defined cc. Scale bar represents the number of allele pairwise differences.

Additional groups were evident, including a cluster of genomes from Poland (n = 7), Germany (n = 7), and Hungary (n = 2), containing eight STs not associated with a cc. Members of this new lineage, while sharing up to five of seven MLST alleles with members of cc174 (lineage 14), were distinct using cgMLST (data not shown). A number of lineages formed larger groups or clades including: lineages 11 and 8; and lineages 2 and 35 with lineage 5. Lineage 11 and lineage 23 contained distinct sub-lineages, (11.1 and 11.2; 23.1 and 23.2) as previously described [19]. The vaccine antigen variants and combinations were associated with particular ccs and lineages, with Cramer’s V values for cc association ranging from 0.458 (NadA) to 0.703 (NHBA) and 0.94 (BAST).

### Lessons learnt

*Neisseria* genomes contain repeat regions, homopolymeric tracts, and paralogous loci [22,23]; all of which may affect sequencing fidelity, assembly synteny, and genome annotation. In addition, whole chromosome extraction and library preparation methods will have downstream effects on the quality of the assembled genome data. A pilot run comprising a minimum of 96 samples is therefore recommended so that the impact of such issues can be ameliorated before protocols are implemented on larger studies, which can be costly. The curation of genomic data within the pubMLST.org/neisseria database was developed using a population annotation approach which places allelic diversity at the centre of analysis [12,13], and therefore required a sequence definition database of proportional size and variability to the organism’s genetic variation. The allelic annotation against a locus specific definition, where accurate start and stop codons were described while capturing the population variability in a meaningful and usable way, requires dedicated manual oversight of curation. Sustaining the computational and comparative value of a genome library will require regular manual checks, data cleaning, and periodic locus definition updates over the life of the database.

## Discussion

The sporadic nature of IMD, combined with the unpredictable occurrence of epidemics and the diversity of meningococci, and especially of meningococcal antigens, makes accurate surveillance an essential element of effective public health measures [24]. The well-documented propensity of particular hyperinvasive lineages to cause IMD outbreaks across continents makes international surveillance of particular importance [7]. Within Europe, there has been coordinated surveillance of IMD since the early 1990s [25]. During this time, meningococcal isolate characterisation was greatly improved by the introduction of molecular methods [26]. The latest enhancement, the use of WGS data was first deployed for routine and comprehensive IMD surveillance on a national scale in the UK, with the establishment of the Meningitis Research Foundation Meningococcus Genome Library (MRF-MGL) from 2010 [19]. The WGS approach provides high-resolution indexing of bacterial diversity potentially replacing phenotypic methods and enabling multiple features of a clinical isolate to be characterised in a single test [13]. With WGS technology becoming increasingly accessible and affordable the EMSC-GL becomes a practical tool for IMD surveillance, complementing and enhancing the information collected by the European Surveillance System (TESSy) database at ECDC and contributing to studies of population biology, evolution, and the consequences of vaccine implementation [27,28].

To maximise international public health impact, the EMSC-GL is hosted within the pubMLST.org/neisseria web-accessible database, which also hosts the MRF-MGL, and allows members to submit genome data and the isolates associated provenance information. This provides publicly-accessible sequence data, described with a uniform nomenclature and linked to relevant provenance and phenotype information. As of early 2017, the continually-expanding pubMLST.org/neisseria database comprised over 2,400 *Neisseria* loci, including 38 sequence-based typing loci such as antigen genes, seven locus MLST, and cgMLST loci. This permits the rapid genetic characterisation and comparison of > 93% of the content of future meningococci genomes added to the genome library with minimal additional curation. Within pubMLST.org/neisseria these loci are grouped into different schemes: currently 40 such curated schemes are publically available, including those for metabolic pathways, plasmid and phage components, typing profiles, and genomic regions [13]. The WGS approach provides a number of practical advantages in addition to its inherent high-resolution and comparability of nomenclature [13,29]. It is a single test that eliminates the multiple testing of a single specimen, which can introduce errors [30]. For example, the incorrect grouping of alleles into a conventional MLST profile can affect the investigation of an outbreak of invasive meningococcal disease [31]. While errors can occur during *de novo* WGS assembly, cgMLST analysis ameliorates these problems as, in a high-quality draft genome, such problems affect a minority of loci which are unlikely to materially alter an analysis [13].

Given its importance in invasion and as a vaccine antigen the meningococcal capsule, which defines the serogroup, is one of the most important isolate characteristics required for disease surveillance. The capsule encoding genome regions are analysable by WGS, resolving anomalies that can arise from phenotypic data and enabling the characterisation of meningococci that do not express capsule. The EMSC-GL contained a small number of isolates with indeterminate W/Y expression and isolates containing genes of more than one capsule type [19]. The difficulty in determining the phenotype of phenotypically W/Y isolates was the result of a rare serine residue at codon position 310 of the NEIS2162 (*csw*) and NEIS2163 (*csy*) genes and, while there is no evidence of multiple capsule expression, horizontal genetic transfer of region A capsule genes has been described for other isolates [14,32]. The presence of different capsule genes highlights recombination potential among closely related meningococci, which can lead to novel phenotypes of clinical and epidemiological significance [14]. Finally, although the production of a capsule is considered to be a virulence factor, there have been reports of un-encapsulated meningococci, one of which was present in the EMSC genomes, causing IMD [33,34].

Analysis of cgMLST data defined 13 lineages, had good ccs concordance as defined by conventional MLST, and identified a new lineage. Vaccine antigens were also rapidly extracted, including those used to assign BAST types, demonstrating a strong association between vaccine antigen variants and cc among the EMSC-GL. This association was consistent with previous studies in other countries such as Ireland, the Netherlands and the UK, and is important when considering the likely impact of protein-based vaccines because prevalent lineages change over time likely causing changes in disease incidence [9,19,35].

The deposition of future meningococcal genome data in the EMSC-GL will permit the resolution of the epidemiology of IMD at the whole genome across the entire continent, and as long as the European Union (EU) maintains funding the infrastructure costs are minimal. The inclusion of additional isolates in close to real time will enable the rapid identification of outbreaks. The past 40 years have seen the spread of successive hyperinvasive meningococci throughout Europe and, while disease levels remain low in most European countries most of the time, epidemics, hyperendemics, and localised disease outbreaks occur regularly across the continent, including transnational outbreaks [19,26,36-38]. The advantage of WGS data is that it comprehensively characterises isolates for their genetic lineage, vaccine antigens, antimicrobial sensitivity, and other properties rapidly and effectively and is able to resolve outbreaks to a high level of discrimination [13,31]. These advantages greatly outweigh perceived high costs of the WGS approach. Future addition of genomes to the library will enable monitoring of secular changes in circulating meningococci over time and an assessment of the possible effects of the introduction of vaccines, such as the protein-based 4CMenB (Bexsero) and LP2086 (Trumenba) vaccines on IMD epidemiology [9]. Effective meningococcal vaccine development and implementation depends on an understanding of the population biology and the effects of herd immunity in an endemic setting, which in turn relies on high-quality surveillance data and isolate characterisation [39]. 

## Conclusion

In conclusion, the detailed meningococcal isolate characterisation available thought the EMSC-GL will be essential for European IMD surveillance, leading to the improvement of control strategies and disease prevention across the continent. This model is generalisable to other bacterial pathogens, many examples can be found on the pubMLST.org website (https://pubmlst.org/databases/).

## *Erratum

In Table 3, the column headings were misaligned. This was corrected on 31 May 2018.
